# Toward Sustainable Polyhydroxyalkanoates: A Next-Gen Biotechnology Approach

**DOI:** 10.3390/polym17070853

**Published:** 2025-03-22

**Authors:** Vipin Chandra Kalia, Rahul Vikram Singh, Chunjie Gong, Jung-Kul Lee

**Affiliations:** 1 Department of Chemical Engineering, Konkuk University, 120 Neungdong-ro, Gwangjin-gu, Seoul 05029, Republic of Korea; vckaliaku@gmail.com (V.C.K.); rahul.negi121@gmail.com (R.V.S.); 2 Cooperative Innovation Center of Industrial Fermentation (Ministry of Education & Hubei Province), Key Laboratory of Fermentation Engineering (Ministry of Education), National “111” Center for Cellular Regulation and Molecular Pharmaceutics, Hubei University of Technology, Wuhan 430068, China; gongcj606@163.com

**Keywords:** polymers, polyhydroxyalkanoates, extremophiles, industrial biotechnology, green technology, environmental sustainability, industrial waste valorization

## Abstract

Polyhydroxyalkanoates (PHAs) are biodegradable biopolymers synthesized by microorganisms and serve as sustainable alternatives to petroleum-based plastics. While traditional PHA production relies on refined carbon sources and pure cultures, high costs and scalability challenges limit commercial viability. Extremophiles, particularly halophiles, have emerged as promising candidates for cost-effective, large-scale production of PHAs. Their ability to thrive in extreme environments reduces contamination risks, minimizes the need for sterilization, and lowers operational costs. Advancements in metabolic engineering, synthetic biology, and CRISPR-based genome editing have enhanced PHA yields by optimizing metabolic flux and cell morphology. Additionally, utilizing alternative feedstocks such as biowaste, syngas, methane, and CO₂ improves economic feasibility. Next-generation industrial biotechnology integrates extremophilic microbes with AI-driven fermentation and eco-friendly downstream processing to enhance scalability. Industrial-scale production of PHAs using *Halomonas* spp. and other extremophiles demonstrates significant progress toward commercialization, paving the way for sustainable biopolymer applications in reducing plastic pollution

## 1. Introduction

Polyhydroxyalkanoates (PHAs) are biodegradable polyesters synthesized by microorganisms as intracellular storage compounds, primarily under environmental stress [1]. The ability of bacteria to withstand stress plays a crucial role in determining their suitability for PHA production. Among the different types of PHA, poly(3-hydroxybutyrate) (PHB) and its copolymers with 3-hydroxyvalerate (3HV) or other monomers significantly influence the material properties, making them suitable for various industrial applications: (i) 3-Hydroxybutyrate (3HB) [CH_3_−CH(OH)−CH_2_−COO^−^] forms poly(3-hydroxybutyrate) (PHB), which is highly crystalline and brittle; (ii) 3-Hydroxyvalerate (3HV) [CH_3_−CH_2_−CH(OH)−CH_2_−COO^−^] is a monomer that reduces crystallinity when incorporated into PHB, improving flexibility and toughness and improving mechanical properties; and (iii) poly(3-hydroxybutyrate-3-hydroxyvalerate (P(3HB-3HV) is a copolymer of PHB and 3HV. The percentage of 3HV (XX mol%) affects polymer properties: (i) higher 3HV content → more flexible, lower melting point, better processability, and (ii) lower 3HV content → stiffer and more brittle, but higher strength. To produce PHBV, a precursor such as propionic acid or valeric acid is fed to microbial culture. These precursors are converted into 3HV units, which in turn influence the copolymer composition (XX mol% 3HV). By adjusting XX mol% 3HV and M.Wt., the polymer’s flexibility, thermal stability, and degradation rate can be fine-tuned. Higher molecular weight results in better mechanical strength, whereas increased 3HV content improves flexibility and reduces brittleness [2].

**Figure 1:**
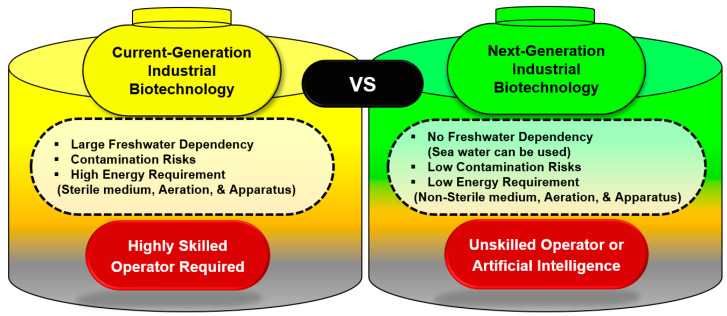
Unique features of current- vs. next-generation industrial biotechnology.

Halophiles and thermophiles offer distinct advantages over mesophiles due to their unique adaptability and physiological traits. Halophiles thrive in high-salinity environments, thereby reducing contamination risks and minimizing the need for sterilization. High salt concentrations facilitate easy PHA extraction, and their salt tolerance allows cultivation in open or semi-open systems, further reducing operational expenses. Thermophiles, thriving at 50–80 °C, enhance substrate solubility, oxygen transfer, and metabolic activity, leading to higher PHA productivity. Their high growth rates reduce fermentation time, and their contaminant resistance simplifies sterilization. Thermophiles also support continuous bioprocessing [3,4,5,6]. Combining halophilic and thermophilic traits could optimize industrial PHA production by enhancing efficiency and reducing costs. Halophiles may be preferable due to their ability to reduce contamination risks without the high energy input required for thermophilic growth. Their open-system cultivation and simple PHA extraction make them more cost-effective. However, thermophiles provide advantages in continuous fermentation and metabolic efficiency. The choice depends on process requirements, but halophiles generally offer greater economic feasibility in large-scale PHA production. These extremophiles offer multiple advantages for sustainable PHA production, most notably by reducing the risk of microbial contamination, which can cause significant economic losses. This forms the conceptual foundation of “Next-Generation Industrial Biotechnology” (NGIB) (Figure 1). It integrates metabolic engineering and synthetic biology to develop cost-effective and sustainable bioprocesses that compete with conventional petroleum-based plastics [7]. Traditional PHA production relies on pure microbial cultures grown on refined carbon sources such as glucose or saccharose. A significant drawback of this approach is the high feedstock cost, which accounts for approximately 40% of total production expenses [8]. Alternative strategies, including biowaste as a cost-effective carbon source, have been explored to mitigate high feed costs. The use of biowastes as feed was successfully demonstrated using heterotrophic microbes [9,10]. In addition to organic substrates, PHA production can leverage inorganic carbon sources such as CO_2_. Photoautotrophic microorganisms, including cyanobacteria, use CO_2_ as a carbon source [11]. Other microbes, such as *Rhodospirillum rubrum*, can metabolize syngas (a mixture of CO, CO_2_, and H_2_) for PHA biosynthesis [12]. Methane (CH_4_) from biogas and natural gas has been identified as a viable substrate for methylotrophic bacteria [13]. Another promising alternative to conventional PHA production is the use of well-defined microbial consortia rather than pure cultures, as this approach eliminates the need for sterilizing the apparatus and medium, and significantly reduces production costs [14]. Using well-defined microbial consortia for PHA production presents several advantages over pure cultures, particularly in reducing sterilization requirements and overall production costs. In traditional PHA production, maintaining pure cultures requires stringent sterile conditions, including the sterilization of apparatus, medium, and bioreactors, which significantly increase energy consumption and operational expenses. In contrast, microbial consortia, composed of naturally coexisting or engineered microbial communities, can outcompete contaminants and function efficiently in nonsterile environments, eliminating the need for costly sterilization processes. Additionally, consortia facilitate efficient substrate utilization by enabling the division of labor among microbial species, leading to improved carbon source conversion and higher PHA yields [6]. They also enhance process resilience by maintaining stability under fluctuating conditions, reducing the risk of culture failure. By leveraging metabolic complementarities and adaptive capabilities, microbial consortia provide a robust and economically viable alternative for large-scale, sustainable PHA production, aligning with NGIB principles. To overcome the limitations of the traditional methods, researchers have proposed NGIB as a novel strategy for cost-effective and sustainable PHA biosynthesis. This concept focuses on utilizing extremophilic microbes as robust microbial chassis for industrial bioprocesses [15,16,17]. PHA production by extremophiles, particularly halophiles, and thermophiles, offers significant advantages as they thrive in extreme environments that naturally suppress microbial contamination, thereby reducing the need for stringent sterilization protocols [4]. This review emphasizes the research efforts that highlight the potential of halophilic microbes in the development of scalable and sustainable PHA production systems. Future efforts should continue to optimize the metabolic pathways and bioprocess conditions to enhance the feasibility of industrial-scale implementation.

## 2. Current Status of Industrial Biotechnology

PHAs are biopolymers synthesized by various microbes and have garnered significant interest because of their biodegradability and potential as sustainable alternatives to petroleum-based plastics. In *Halomonas*, the primary biosynthetic pathway leading to scl-PHA synthesis involves the condensation of two acetyl-CoA molecules by 3-ketoacyl-CoA synthase (PhaA). The resultant acetoacetyl-CoA is then reduced by acetoacetyl-CoA reductase (PhaB) using NAD(P)H. The R-3-hydroxybutyryl-CoA produced in the second step is polymerized into P(3HB) by PHA synthase. For mcl-PHA synthesis, 3-hydroxy acyl-CoA precursors are derived from fatty acid de novo synthesis or β-oxidation of fatty acids [6]. PHA production involves multiple stages, including microbial screening, strain improvement, laboratory-scale optimization, and large-scale industrial implementation. Despite advancements in genetic engineering and industrial biotechnology, several challenges persist, particularly in terms of production costs and scalability [18]. The initial step in PHA production involves identifying microbial strains capable of efficiently synthesizing PHAs. Wild-type bacterial strains were first used, followed by genetic engineering to enhance production. The production of short-chain-length polyhydroxyalkanoates (scl-PHA) was successfully demonstrated using *Ralstonia eutropha* and *Alcaligenes latus* [19]. Additionally, copolymers such as poly(3-hydroxybutyrate-co-3-hydroxyhexanoate) have been synthesized using *Aeromonas hydrophila* and *R. eutropha* [14,20]. Various species of *Pseudomonas* produce medium-chain-length PHA (mcl-PHA). Enhancing production through strain improvement has been achieved by deleting genes involved in the β-oxidation pathway. This genetic modification enables *Pseudomonas* spp. to maintain their chain length and structure, producing homopolymers, copolymers, and functionally modified PHAs through the selective addition of fatty acids [21]. *Escherichia coli*, which does not naturally produce PHAs, has been engineered to become a hyperproducer of various PHA types [22,23]. *E. coli*, despite its inability to naturally synthesize PHAs, has been extensively engineered to become a next-generation microbial chassis for PHA production due to its well-characterized genetics, rapid growth rate, and ease of genetic manipulation. Key PHA biosynthetic pathways have been successfully introduced into *E. coli* through metabolic engineering, enabling the production of PHA copolymers. Advanced genetic tools, such as CRISPR/Cas9, synthetic biology, and metabolic flux optimization, have further enhanced *E. coli*’s PHA production capacity by improving precursor supply, balancing redox states, and eliminating competing pathways. Advances in industrial biotechnology have enabled *E. coli* and *R. eutropha* to achieve high cell densities and productivity, reaching dry biomass levels of 232 g/L and productivity of 4.63 g/L/h, particularly when recombinant *E. coli* was engineered using the *phaCAB* operon from *A. latus* [24,25,26]. Despite advancements in strain engineering and bioprocess optimization, several limitations remain. High production costs, ranging from USD 4 to 6 per kg, make PHAs less competitive than fossil-fuel-derived plastics [27,28,29]. The key challenges include expensive sterilization processes, prevention of contamination, low feed-to-PHA conversion ratios, and high energy consumption for aeration. Additionally, the reliance on glucose as the primary feedstock presents economic and environmental concerns, including substantial water usage and costly downstream processing requirements [17]. Future research should focus on overcoming the challenges related to fermentation technology, alternative feedstock utilization, and cost-effective downstream processing methods, which are crucial for achieving commercially viable PHA production.

## 3. Next-Generation Industrial Biotechnology

The effort to enhance industrial-scale PHA production has increasingly focused on utilizing extremophiles. These microorganisms can thrive under harsh physiological conditions that are inhospitable to most bacteria, making them ideal candidates for bioprocessing [30,31,32,33,34,35,36,37,38]. By leveraging extremophiles, PHA production can be made more sustainable and cost-effective. The ability of extremophiles to withstand high temperatures and salinity significantly restricts microbial contamination, thereby reducing the need for sterilization and minimizing energy consumption [39,40,41,42,43]. This selective growth advantage allows for more controlled and efficient fermentation. Industrial PHA production incorporates continuous culture, low-energy processing, and automated control systems to enhance its efficiency. These measures reduce operational costs while improving yield and quality [15,17,44]. Economic and environmental sustainability are being addressed through the use of alternative feedstocks, including hydrolysates of cellulosic materials, pretreated sludge, kitchen waste, and syngas [45]. Substituting freshwater with seawater or treated wastewater is a cost-effective and sustainable solution for PHA production. High-temperature and high-salinity conditions reduce the need for sterilization and lower energy requirements. Traditional stainless-steel fermenters may also be replaced by more cost-effective materials, such as ceramics, plastics, or cement, making PHA production more economically viable. Extremophiles and recombinant bacteria serve as an efficient biological chassis for the optimized synthesis of biopolymers [15,30,46].

## 4. PHA Production by Halophilic Archaea

Among the various microorganisms known to accumulate PHAs under hypersaline conditions, a few notable examples include archaea such as *Haloarcula* sp., *Haloferax* sp., *Halogeometricum* sp., *Halopiger* sp., and *Natrinema* sp.

### 4.1. PHA Production from Pure Sugars

Initial studies on PHA production by archaeal species used pure sugars as feed under highly saline conditions. A study by Han et al. demonstrated that *Haloarcula marismortui* accumulates poly(3-hydroxybutyrate) [P(3HB)] up to 21% of its cell dry weight (CDW) when grown on glucose and 20% NaCl under shake-flask conditions [47]. *Haloferax mediterranei* fermented glucose and yeast extract to accumulate P(3HB-3HV) in a fed-batch culture. After 117 h, the PHA yield reached 41.69 g/L. The resulting copolymers had two distinct molecular weight (M.Wt.) distributions: (i) 93.4 wt% with 10.7 mol% 3HV and M.Wt. of 569.5 kg/mol and (ii) 6.6 wt% with 12.3 mol% 3HV and M.Wt. of 78.2 kg/mol [48]. *H. mediterranei* CGMCC 1.2087 synthesized P(3HB-3HV) up to 24% and 18% (wt/wt) in limited and nutrient-rich media, respectively, with 1% starch as the carbon source [49]. The archaeon *Halogranum amylolyticum* TNN58 synthesizes P(3HB-3HV) with a 3HV content of 20.1 mol% when cultivated with glucose as the carbon source. Process optimization, especially medium and culture conditions, enabled an 8-fold increase in PHBV production and a 4-fold increase in CDW in a fed-batch process compared to a batch process, making it a strong candidate for large-scale PHA production [50]. *Halogeometricum borinquense* strain E3, an extremely halophilic archaeon, achieved PHA accumulation up to 73.51% when supplemented with 2% glucose. Adding a precursor enabled the biosynthesis of P(3HB-3HV) containing 21.47 mol% 3HV [51]. Another extremophilic archaeon, *Natrinema ajinwuensis* RM-G10, demonstrated PHA accumulation of 61.02% with a 3HV content of 13.93 mol% when grown under optimized conditions [52].

### 4.2. PHA Production from Biowastes

PHA production by halophiles using starch-based feedstock carbon sources has been reported previously (Table 1). *H. mediterranei* accumulated 50.8% P(3HB-3HV) of CDW from extruded starch under high salt concentrations during fed-batch fermentation [53]. Using extruded rice bran and corn starch (1:8 g/g) as carbon sources, *H. mediterranei* achieved a PHA yield of 77.8 g/L in a 5 L repeated fed-batch fermenter, with sustained long-term production under hypersaline conditions [54]. *Natrinema* sp. strain 1KYS1 accumulated 0.055 and 0.075 g/L PHA when grown on starch and corn starch, respectively [46]. Dairy and ethanol industry waste has been used as a cheaper feedstock for *H. mediterranei* DSM 1411, which produces 50% P(3HB-3HV) from hydrolyzed whey, even without precursors. In contrast, *Pseudomonas hydrogenovora* and *Hydrogenophaga pseudoflava* required valeric acid supplementation to yield comparable copolymers [55]. The same strain synthesized (i) 72.8% P(3HB-3HV) (6 mol% 3HV) from sugars from hydrolyzed whey and (ii) 87.5% PHA terpolyester (21.8 mol%-3HV and 5.1 mol%-4HB) from precursor-supplemented whey sugars [56]. *H. mediterranei* DSM 1411 used vinasse (ethanol industry waste) pretreated with activated carbon (25–50% *v*/*v*) to yield 13.79 g/L PHA while simultaneously reducing pollution, biological oxygen demand (BOD_5_) by 78%, and chemical oxygen demand by 80% [57]. Further studies on rice-based ethanol waste resulted in 71% P(3HB-3HV) (15.4 mol% 3HV) of CDW, with an 83% pollution load reduction and 96% recovery of medium salts, enhancing the industrial feasibility of the process [58]. *H. marismortui* metabolized 10% raw vinasse into 2.8 g/L P(3HB) in shake flasks, whereas activated carbon 100% pretreated vinasse led to 4.5 g /L P(3HB) [59]. *Natrinema* sp. strain 1KYS1 accumulated 0.091 g/L PHA, when grown on whey [46]. In addition, PHA production by Haloarchaea has been reported in agro-industrial waste and other feedstocks. *Natrinema* sp. strain 1KYS1 was reported to have the potential to accumulate 0.039, 0.046, and 0.077 g/L PHA in CDW when grown on apple waste, melon waste, and tomato waste, respectively [46]. Phenolic compounds are inhibitory to bacterial growth. *H. mediterranei* used 15% olive mill wastewater as feedstock at 22% salt concentration, yielding 0.2 g/L PHA with a 43% CDW content. Without precursor supplementation, P(3HB-3HV) had 6.5 mol% 3HV, with lower melting points (140.1 °C and 154.4 °C) compared to pure P(3HB), eliminating the need for costly dephenolization [60]. *H. borinquense* strain E3 utilized sugarcane bagasse (SCB) hydrolysate to produce (i) 50.4% P(3HB-3HV) (13.29 mol% 3HV) from 25% SCB hydrolysate, and (ii) 45.7% P(3HB-3HV) from 50% SCB hydrolysate [61]. The same strain metabolized cassava waste and starch to yield (i) P(3HB-3HV) (13.11 mol% 3HV) at 4.6 g/L from starch, and (ii) P(3HB-3HV) (19.65 mol% 3HV) at 1.52 g/L from cassava waste, addressing environmental concerns related to industrial cassava discharge [62].

Haloarchaea, particularly *H. mediterranei*, offers a sustainable and cost-effective approach for PHA production by utilizing diverse low-cost feedstocks. Their ability to thrive in high-salt environments eliminates the need for stringent sterilization, which can potentially reduce production costs. However, environmental concerns regarding salt disposal must be addressed, as the discharged effluent often contains total dissolved solids above 2000 mg/L [58]. Future research should focus on optimizing bioprocessing conditions and developing salt recovery strategies to enhance industrial feasibility.

## 5. PHA Production by Halophilic Bacteria

Among halophiles, several bacterial genera, including *Halomonas*, *Oceanimonas*, *Salinivibrio*, *Bacillus*, and *Vibrio* spp. have been extensively studied for PHA production.

### 5.1. PHA Production from Sugars

P(3HB) was produced by alkalophilic and halophilic *Halomonas* sp. KM-1 under non-sterile batch culture conditions. The fermentation yielded 7.97 g/L PHB after 24 h in a 5% glucose medium. Increasing glucose concentration to 10% enhanced P(3HB) yield to 26.3 g/L at 36 h. In contrast, *Halomonas* sp. O-1 produced only 2.13 g/L P(3HB), whereas *Halomonas* sp. KM-1 produced 28.34 g/L, demonstrating strain-dependent genetic variability [63]. Fed-batch culture studies of *Halomonas boliviensis* further illustrated the influence of the medium components on P(3HB) synthesis. Optimal growth conditions with NH_4_Cl and K_2_HPO_4_ supplementation resulted in a 5.89-fold higher cell biomass than 0.1% glutamine alone, with a resultant PHB yield of 20.7 g/L within 18 h. Further optimization using MSG, NH_4_Cl, and K_2_HPO_4_ at specific concentrations yielded 35.64 g/L PHB [64]. *Halomonas* sp. YLGW01 produced 8.65 g/L P(3HB) using fructose as the carbon source [65]. Advancements in genetic engineering have further improved PHA production. In *Halomonas bluephagenesis* TD01, engineering efforts targeting phasin (PhaP) genes have influenced PHA granule size. In contrast, overexpression of *minC* and *minD* genes (coding for Z-ring positioning protein) resulted in large cell sizes with PHA granules up to 10 μm, indicating a correlation between cell morphology and PHA accumulation [66]. Additionally, bacterial morphology regulation via *mreB* (encoding a dynamic cytoskeletal protein) and *ftsZ* (encoding a bacterial fission ring formation protein) was leveraged in *Halomonas campaniensis* LS21 using temperature-sensitive plasmids. Controlled expression at 37 °C increased PHB yield by 80% while facilitating cell harvesting and downstream processing [67]. Halophilic *Halomonas profundus* grows optimally at pH 8–9 and 2–3% NaCl, producing P(3HB) and P(3HB-3HV) [68]. Continuous culture studies demonstrated sustained PHA production by *Halomonas* TD01, achieving 24 g/L PHA in a 14-day unsterile process. Under nitrogen-limiting conditions, PHB content increased to 65–70%, but the PHA yield was lower at 13 g/L due to the medium dilution in the second fermenter [69]. Genetic modifications in *Halomonas* TD01, including the deletion of PHA depolymerase and overexpression of genes involved in propionic acid metabolism, yielded P(3HB-3HV) with 12 mol% 3HV [70]. Further enhancements using the LacIq-Ptrc system in *Halomonas* TD08 increased the PHB content from 69 to 82 wt%, facilitating downstream processing and reducing production costs [71].

Clustered regularly interspaced short palindromic repeat interference (CRISPRi) technology has been applied to *Halomonas* species TD01 to regulate genes such as *ftsZ*, *prpC*, and *gltA*. This led to elongated cell morphology, enhanced acetyl-CoA flux toward PHA synthesis, and increased P(3HB-3HV) copolymer production of up to 13 mol% 3HV [72]. Several additional *Halomonas* spp. have demonstrated significant PHA production capabilities under varied conditions: (i) *H. elongata* strains accumulated PHA at 10% NaCl with diverse carbon sources, *H. elongata* A1 synthesized P(3HB) at 2.59 g/L and 0.49 g/L using glucose and carboxymethyl cellulose, respectively [73,74]; (ii) *H. venusta* KT832796 optimized carbon-to-nitrogen ratios to achieve an 8.65-fold increase in PHA production [75]; (iii) *H. cupida* J9 produced short- and medium-chain-length PHAs (scl-co-mcl PHAs) from glucose and glycerol in unsterile fermentation [76]; (iv) *H. pacifica* ASL10 and *H. salifodiane* ASL11 produced P(3HB-3HV) from sucrose with yields of 6.9 g/L and 7.1 g/L at a pH of 7 and 1.7% NaCl [77]; a few other strains, such as *Oceanimonas* strain GK1 accumulated 75 wt% P(3HB) at 5% NaCl with sucrose and peptone as carbon and nitrogen sources [78]; (v) *Salinivibrio* sp. TGB10 achieved high PHB and PHBV yields from various sugars, with propionate supplementation enhancing PHBV content to 72.02 mol% 3HV [79]; and (vi) *Vibrio proteolyticus* strain produced 2.7 g/L PHA on M9 minimal medium supplemented with fructose (2% *w*/*v*) as carbon source in a NaCl (5% *w*/*v*). PAH copolymer P(3HB-3HV) with a 3HV content of 15.8 mol% was produced by the addition of propionate (0.3%) to the medium, even under unsterile conditions and higher NaCl concentrations [80].

### 5.2. PHA Production from Industrial and Agricultural Wastes

Several species of *Halomonas* and *Bacillus* have shown promising PHA production capabilities (Table 2). *Halomonas campisalis* MCM B-1027 produced 0.36 g/L PHA (5.6 mol% 3HV) on 1% (*v*/*v*) aqueous extract of bagasse [81]. *H. halophila* accumulated up to 3.26 g/L P(3HB), with NaCl concentrations regulating P(3HB) yields from hydrolysates of cheese whey, corn stover, sawdust, sugar beet, and spent coffee grounds. In contrast, with pure sugars such as cellobiose, galactose, and glucose as carbon sources, PHA yields by *H. halophila* were 2.59, 3.41, and 4.58 g/L [82]. Three recombinant strains (*H. elongata* P2) reached P(3HB) contents of 0.44 g/L, 1.28 g/L, and 1.81 g/L when cultivated on wheat straw, mixed substrates, and oleic acid, respectively [74]. Under unsterilized conditions using fructose syrup, the P(3HB) content by *Halomonas* sp. YLGW01 increased to 95.26%. The strain exhibited enhanced cell size (8.39 μm) compared to 2.34 μm on glucose, facilitating downstream processing and polymer recovery [65]. The halophilic bacterium *Bacillus megaterium* uyuni S29 metabolizes desugarized sugar beet molasses (a high-salinity effluent) to produce 10.02 g/L P(3HB) under batch cultivation, achieving a 2- to 3-fold increase in biomass [35]. *B. megaterium* also metabolizes desugarized sugar beet molasses, yielding 1.2 g/L P(3HB) across six cultivation cycles [83].

*Halomonas* TD01 was genetically modified by introducing the conjugative plasmid, pSEVA341, using the LacIq-Ptrc system to induce gene expression. The deletion of 2-methyl citrate synthase and PHA depolymerase resulted in the *Halomonas* TD08 strain producing P(3HB-3HV) (4–6 mol% 3HV). Overexpression of threonine synthesis and threonine dehydrogenase improved the PHA yields. The inhibition of cell division using MinCD led to 1.4-fold longer cells, enhanced PHB accumulation from 69 to 82 wt%, simplified downstream processing, and reduced production costs [71]. *Halomonas* sp. KM-1 successfully utilized glycerol-rich biodiesel industry waste to produce P(3HB) [84]. *Halomonas desertis* G11 synthesized PHA copolymer using biodiesel-derived glycerol [85]. *Halomonas taeanenisis YLGW01* metabolized crude glycerol to produce P(3HB-3HV) (17 mol% 3HV). Optimization and activated carbon treatment improved PHA yields to 10.5 g/L during fed-batch fermentation [86]. *Halomonas hydrothermalis (MTCC 5445)* accumulates PHA using glycerol and peptone at high salt concentrations, achieving 2.61 g/L yield in shake flasks [87]. *Bacillus sonorensis* SM-P-1S and *Halomonas hydrothermalis* SM-P-3M accumulated 0.2 g/L and 0.3 g/L P(3HB), respectively, from *Jatropha* biodiesel byproducts, reducing production costs [88].

PHA production under extreme conditions by various *Halomonas* spp. has been quite successful: (i) *Halomonas cupida* J9 produced scl-co-mcl PHA ((3HB-3-hydroxydodecanoate, 3HDD) from glucose and glycerol under unsterile conditions, showing superior thermal and mechanical properties [76]; (ii) *Halomonas alkaliantarctica* synthesized P(3HB-3HV) from crude glycerol without additional precursors, demonstrating substrate-independent PHA yields [89]; and (iii) *Halomonas daqingensis* effectively converted crude glycerol from algal biodiesel waste residues into PHA, achieving 0.236 g/L PHA under mesophilic conditions with 5% NaCl [90]. Enhancing PHA yield with precursors and fermentation strategies: (i) *Halomonas hydrothermalis* synthesized P(3HB-3HV) with 50.15 mol% 3HV using valerate as the precursor; PHA yields and molecular weight declined with an increase in NaCl concentration from 40 g/L to 100 g/L [91]; and (ii) *H. bluephagenesis TD40* efficiently produced P(3HB-co-4HB) in industrial-scale (1–5 m³) bioreactors under non-sterile conditions. Waste gluconate reduced feed costs by 60%, achieving 60.4 g/L PHA (13.5 mol% 4HB) within 36 h. Yield improved to 74% by decreasing waste corn steep liquor usage, reducing energy consumption during downstream processing [92]. Other halophilic bacteria, including *Yangia* sp. ND199 produced P(3HB-3HV) in the presence of 4.5% NaCl. The highest PHBV productivity (53.2 wt% with 2.9 mol% 3HV) was obtained on crude glycerol supplemented with yeast extract. Further enhancement (56 wt% PHA, 0.61 g/L/h productivity) was achieved using high-fructose corn syrup [93].

Advances in PHA synthesis using halophilic microorganisms have demonstrated significant potential for the sustainable production of biopolymers. Genetic modifications, efficient carbon source utilization, and process optimization have enhanced the PHA yields and reduced production costs. The ability of these microbes to grow under non-sterile, high-salinity conditions further supports their industrial scalability. Integrating waste-derived feedstocks such as crude glycerol, biodiesel byproducts, and lignocellulosic hydrolysates mitigates environmental waste and improves economic feasibility. Future research should optimize the fermentation conditions, refine downstream processing, and explore novel extremophiles for diversified PHA compositions and enhanced properties.

**Table 2:** Diversity of halophilic bacteria to produce polyhydroxyalkanoates.

PHA Producer	Substrate (%)	Salt (NaCl) (%)	Reactor Condition	Cell Dry Mass (g/L)	Polyhydroxyalkanoate (PHA)			References
					Composition (%mol Copolymer)	Content (wt%)	Yield (g/L)	
Pure Sugars								
*Halomonas* sp. KM-1	Glucose (10)	0.1	Batch	38.4	P(3HB)	73.7	28.34	[63]
*Halomonas* sp. O-1	Glucose (10)	0.1	Batch	6.9	P(3HB)	31	2.13	[63]
*Halomonas boliviensis*	Glucose (2)	4.5	Shake flask (Fed-batch)	44	P(3HB)	81	35.4	[64]
*Halomonas bluephagenesis* TD01	Glucose (3)	5	Shake flask	12.88	P(3HB)	76.16	9.81	[66]
*Halomonas campaniensis* LS21	Glucose (1.5)	4	Shake flask	27	P(3HB)	30	8.1	[67]
*Halomonas profundus* AT1214	Glucose (1)	2.7	2-stage batch (5 L)	-	P(3HB)	-	0.27	[68]
	Glucose ^a^ (1)	2.7	2-stage batch (5 L)	-	P(3HB-3HV) [28 mol% 3HV]	-	0.27	
*Halomonas* sp. TD01	Glucose (3)	6	Continuous culture	40	P(3HB)	60	24	[69]
	Glucose (3)	6	Continuous culture (500 L)	112	P(3HB)	70	78.4	[66]
	Glucose ^a^ (3)	6	Continuous culture (500 L)	80	P(3HB-3HV) ^a^ [12 mol% 3HV]	70	56	[70]
	Glucose (3)	6	Shake flask	10.22	P(3HB)	77.68	7.94	[72]
*Halomonas* sp. TD08 pSEVA341) (blank vector)	Glucose	6	Shake flask	8.61	P(3HB-3HV) [trace 3HV]	70.45	6.05	[71]
*Halomonas* sp. TD-*gltA*2 (Rec.)	Glucose (3)	6	Shake flask	13.53	P(3HB)	71.77	9.71	[72]
*Halomonas halophila* CCM 3662	Glucose (2)	6.6	Shake flask	5.62	P(3HB)	81.5	4.58	[82]
	Cellobiose (2)	6.6	Shake flask	2.86	P(3HB)	90.8	2.59	
	Galactose (2)	6.6	Shake flask	4.22	P(3HB)	80.7	3.41	
*Halomonas elongata* 2FF	Glucose (1)	10	Shake flask	-NR	P(3HB)	-NR	0.4	[73]
*H. elongata* A1	Glucose (1)	5	Shake flask	6.75	P(3HB)	22.81	2.59	[74]
	Cellulose (1)	5	Shake flask	4.17	P(3HB)	11.80	0.49	
*Halomonas venusta* KT832796	Glucose (2)	1.5	Single pulse feeding	37.9	P(3HB)	88.12	33.4 (8.65-fold)	[75]
	Glucose (2) ^b^	1.5	Fed-batch (2 L)	3.52	P(3HB)	70.56	2.48	
*Halomonas cupida* J9	Glucose ^a^	8	Shake flask	5.5	P(3HB-3HDD)	32	1.76	[76]
*Halomonas pacifica* ASL10	Sucrose (2) + Ammonium sulphate (0.2)	(up to 170)	Shake flask	9.59	P(3HB-3HV)	71.9	6.9	[77]
*Halomonas salifodiane* ASL11	Sucrose (2) + Ammonium sulfate (0.2)	(up to 170)	Shake flask	8.86	P(3HB-3HV)	80.1	7.1	[77]
*Vibrio proteolyticus*	Fructose (2)	5	Shake flask	4.94	P(3HB)	54.7	2.7	[80]
	Fructose ^a^ (2)	5	Shake flask	3.62	P(3HB-3HV) [15.8 mol% 3HV]	47.68	1.73	
Industrial and Agricultural Wastes								
*Halomonas campisalis* MCM B-1027	Bagasse (1)	4.5	Shake flask	0.78	P(3HB-3HV) [5.6 mol% 3HV]	46.5	0.36	[81]
	Banana peel (1)	4.5	Shake flask	0.53	P(3HB-3HV) [52.04 mol% 3HV]	10.5	0.37	
	Orange peel (1)	4.5	Shake flask	0.92	P(3HB-3HV) [52.04 mol% 3HV]	21.5	0.19	
*H. halophila* CCM 3662	Cheese whey hydrolysate	6.6	Shake flask	8.50	P(3HB)	38.32	3.26	[82]
	Molasses	6.6	Shake flask	4.05	P(3HB)	64.06	2.57	
*H. elongata* P2	Wheat straw	5	Shake flask	8.42	P(3HB)	5.19	0.44	[74]
	Mixed substrates	5	Shake flask	7.76	P(3HB)	16.49	1.28	
	Oleic acid	5	Shake flask	5.76	P(3HB)	27.42	1.81	
*Halomonas* sp. YLGW01	Fructose syrup (2)	2	Shake flask	9.15	P(3HB)	94.62	8.65	[66]
*Bacillus megaterium* uyuni S29	Sugar beet molasses (1)	1	Shake flask	16.7	P(3HB)	60	10.02	[35]
	Sugar beet molasses (5)	0/5	Pilot scale (500 L)	20.4	P(3HB)	58.8	12	[83]
*Halomonas* TD08 (pSEVA341-thrACBilvA) ^c^	Glycerol	6	Shake flask	6.65	P(3HB-3HV) [6.12 mol% 3HV]	67.14	4.46	[71]
*Halomonas* sp. KM-1	Pure glycerol (2)	-	Shake flask	4.69	P(3HB)	40.5	1.9	[84]
*Halomonas* sp. KM-1	Pure glycerol (5)	-	Shake flask	5.13	P(3HB)	44.8	2,3	[84]
*Halomonas* sp. KM-1	Waste glycerol (3)	-	Shake flask	4.10	P(3HB)	39.0	1.6	[84]
*Halomonas desertis* G11	Glycerol (1)	5	Shake flask	2.29	P(3HB-3HV) [52 mol% 3HV]	68	1.54	[57]
*Halomonas cupida* J9	Glycerol	10	Shake flask	3.5	P(3HB-3HDD)	29	1.01	[76]
*Halomonas* sp. YLGW01	Glycerol (2)	2	Shake flask	17.5	P(3HB-3HV) [13 mol% 3HV]	60.0	10.5	[86]
*Halomonas hydrothermalis* MTCC5445	Glycerol (5) (+Peptone)	3.5	Batch	-	P(3HB)	-	2.59	[87]
	Glycerol (3) (+Peptone)	3.5	Batch	-	P(3HB)	-	2.61	
*H. hydrothermalis* SM-P-3M	Jatropha biodiesel byproducts (2)	0.5	Batch	0.40	P(3HB)	75.8	0.30	[88]
*Bacillus sorensis* SM-P-1S	Jatropha biodiesel byproducts (2)	0.5	Batch	0.283	P(3HB)	71.8	0/20	[88]
*Halomonas alkaliantarctica* DSM 15686	Biodiesel-derived glycerol (85%) (1)	1.94	Shake flask	-	P(3HB-3HV) [2.77 mol% 3HV]	11	3.5 ^b^	[89]
	Biodiesel-derived glycerol (85%) (5)	1.94	Shake flask	-	P(3HB-3HV) [1.82 mol% 3HV]	18	5.8 ^b^	
	Biodiesel-derived glycerol (85%) (8)	1.94	Shake flask	-	P(3HB-3HV) [1.65 mol% 3HV]	9	3.0 ^b^	
*Halomonas daqingensis*	Algal biodiesel waste residue (Crude glycerol) (3)	3.5	Batch	0.362	P(3HB-3HV)	65.2	0.236	[90]
*Salinivibrio* sp. M318	Glycerol + waste fish oil and sauce (as nitrogen source) (3)	4.5	Fed-batch	5.7	P(3HB), P(3HB-3HV), P(3HB-4HB) ^a^ [2.9 mol% 3HV]	52.8	3	[91]
*Halomonas organivorans* CCM 7142	Waste frying oil (2)	4	Shake flask	3.64	P(3HB)	61.98	2.26	[91]
*Halomonas hydrothermalis* CCM 7104	Waste frying oil (2)	8	Shake flask	1.60	P(3HB)	23.76	0.38	[91]
	Waste frying oil (2)	8	Shake flask	2.75	P(3HB-3HV) ^a^ [7.16 mol% 3HV]	47.17	1.29	
*Halomonas neptunia* CCM 7107	Waste frying oil (2)	6	Shake flask	2.28	P(3HB)	55.71	1.27	[91]
	Waste frying oil (2)	8	Shake flask	1.23	P(3HB-3HV) ^a^ [26.07 mol% 3HV]	15.85	0.19	
*Halomonas bluephagensis* TD01	Glucose (3) (60MMG medium with γ-butyrolactone + waste corn steep liquor; waste gluconate)	6	Continuous culture (Pilot:5 m^3^)	100	P(3HB-4HB) [13.5 mol% 4HB]	60	0.6	[92]

a: Precursor added; b: Ammonium citrate (0.1); c: (*thrACB* operon and *ilvA* gene coding for threonine dehydrogenase). -NR: Not reported

Based on a perusal of the key observation made on archaeal and bacterial capacities to metabolize different feedstocks and their PHA yielding capacities considering both PHA content (%) and PHA concentration (g/L), the following conclusions can be drawn (Table 3):

- Archaeal feedstocks generally yield much higher PHA concentrations (up to 77.8 g/L) than bacterial feedstocks (max 10.5 g/L).
- Bacterial systems achieve higher PHA content (95.26%) than archaea (max 87.5%).
- Crude glycerol and sugar beet molasses appear to be the best bacterial feedstocks for industrial feasibility, while rice bran–corn starch mix and hydrolyzed whey dominate archaeal production.
- Waste valorization is a key advantage in both cases, although archaea demonstrate broader substrate adaptability.
- Archaea are better suited for large-scale biopolymer production, while bacteria are more cost-effective in non-sterile industrial settings.

## 6. Robust Contender for PHA Production

While traditional bacterial candidates such as *Pseudomonas, Bacillus*, and *Ralstonia* have been extensively studied for PHA production [94,95], recent research has shifted the focus toward extremophiles, particularly *Halomonas* spp. (Table 4). This genus can produce PHAs in bulk at a low cost, making it a promising candidate for NGIB.

- *Halomonas* spp. as an optimal chassis for PHA production: *Halomonas* species, particularly *H. bluephagenesis* and *H. campaniensis*, have been identified as highly suitable hosts for PHA biosynthesis due to their fast growth, high tolerance, and ease of genetic manipulation [69,96]. These bacteria can thrive under unsterile conditions, with *H. campaniensis* reported to sustain growth in artificial seawater for over two months [32,97,98].
- Genetic engineering strategies: Recent advancements in the genetic modification of *Halomonas* spp. involve the development of unique plasmids carrying inducible gene expression systems controlled by constitutive promoters [99,100,101]. Tools like CRISPR/Cas9 and CRISPRi have been employed to regulate metabolic flux, particularly the nicotinamide adenine dinucleotide hydride/nicotinamide adenine dinucleotide (NADH/NAD+) ratio, by deleting flavoprotein genes to enhance PHA synthesis in *H. bluephagenesis* [13,102,103,104,105]. Additionally, *H. bluephagenesis* has been engineered to optimize oxygen uptake under low oxygen conditions via inducible promoter control [106].
- Enhancing PHA production through morphological and metabolic engineering: Genetic modifications, including the overexpression of *sulA* (encoding cell division inhibitor protein) or *minCD* and deletion of *mreB*, have resulted in higher cell densities [1,67,107,108]. In addition to the downstream processing techniques, these strategies promote the formation of larger cells containing larger PHA granules [66]. Moreover, self-flocculation mechanisms have been optimized to facilitate easy harvesting [109]. Synchronizing cell autolysis with substrate exhaustion has also been explored to improve the cost efficiency of PHA synthesis [110].
- Industrial-scale achievements and economic feasibility: Studies have successfully produced PHA copolymers containing monomers, such as 3HB, 4HB, HHx, and functionally modified 3-hydroxyhex-5-enoate (HHxE) [92,111,112]. This has enabled industrial-scale PHA production, reaching biomass levels of 100 g/L and copolymer yields of 60 wt% in 5000 L reactors [92,113]. With these next-generation biotechnological advancements, the production cost of PHA is projected to decrease. The cost of P(3HB-co-4HB) production by genetically engineered *H. bluephagenesis* was reported to be USD 1.98 /kg by replacing γ-butyrolactone with unrelated carbon sources. The production cost was envisaged to be reduced further to 1.66–1.94 /kg for slaughtering waste streams. It was also observed that replacing glucose with gluconate can help to achieve a USD 0.75/kg cost-reduction [88].

**Figure 2:**
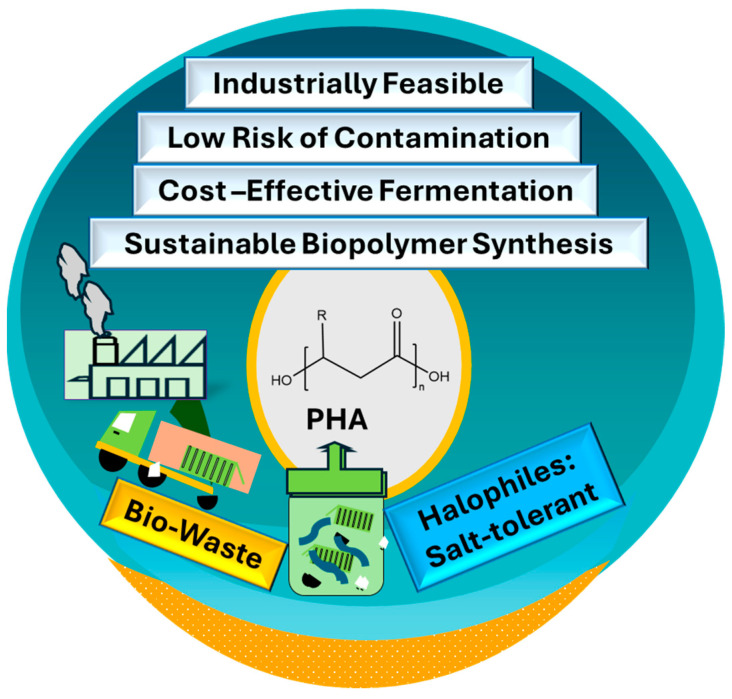
The significance of extremophiles for producing polyhydroxyalkanoates.

The shift toward *Halomonas* spp. for PHA production represents a breakthrough in industrial biotechnology. These extremophiles have demonstrated remarkable potential for cost-effective large-scale PHA production through genetic modifications and optimized process parameters (Figure 2). Integrating advanced genetic tools and sustainable cultivation techniques is expected to drive the commercialization of PHAs as viable alternatives to petroleum-based plastics. Enhancing PHA recovery from extremophiles, particularly halophiles, requires modifications to conventional extraction methods. Due to their high intracellular osmotic pressure, extreme halophiles lyse easily in hypotonic conditions, eliminating the need for harsh chemical, enzymatic, or mechanical disruption. Optimization involves controlled osmotic shock using deionized water and centrifugation to recover intact PHA granules. Reducing salt contamination and improving granule purity may require additional washing steps. Moreover, scalable strategies should focus on minimizing dilution effects and optimizing biomass harvesting from high-salinity cultures. Integrating eco-friendly extraction processes can enhance yield, reduce costs, and improve the sustainability of PHA production [6,40,114].

**Table 4:** *Halomonas* as a promising microbe for sustainable polyhydroxyalkanoate production.

Distinctive Traits	References
Extremophilic bacteria with high salt tolerance and higher oxygen uptake, even at extremely low oxygen levels, are suitable for industrial PHA production	[32,69,92,96,98,106]
Microbes engineered with a constitutive promoter-mediated genetic system	[99,100,101,102]
Modified cell shape (filamentous) with higher density, larger PHA granules for easy recovery—downstream processing achieved by deleting gene *(mreB*) in association with overexpression of genes of cell division (*minCD* or *sulA*)	[66,67,107,108,109]
CRISPR/Cas9 and CRISPRi-based multi-purpose PHA synthase and manipulating carbon flux and energy regulation (NADH/NAD+), enhanced acetyl-CoA diversion leading to enhanced PHA yield	[13,103,104,105]
Diversity of PHA copolymers with monomers of 3-hydroxybutyrate, 4-hydroxybutyrate, and 3-hydroxy hexanoate	[89,111,112]
Commercial level PHA production: 5000 L reactors	[92,113]

PHA: polyhydroxyalkanoate; CRISPR/Cas9: Clustered regularly interspaced short palindromic repeats Cas protein systems; CRISPRi: Clustered regularly interspaced short palindromic repeats interference; NADH/NAD+: Nicotinamide adenine dinucleotide hydride/Nicotinamide adenine dinucleotide; *mreB* (encoding dynamic cytoskeletal protein); *minC* and *minD* (encoding Z-ring positioning protein); *sulA* (encoding cell division inhibitor protein).

## 7. Perspectives

Advancing NGIB for PHA production requires research across multiple areas. Metabolic engineering and synthetic biology focus on enhancing metabolic pathways to improve PHA yield and monomer composition, utilizing CRISPR/Cas-based genome editing to fine-tune regulatory networks, and engineering robust microbial chassis for efficient biosynthesis. Extremophile-based fermentation leverages halophilic and thermophilic bacteria for cost-effective, unsterile production while developing adaptive evolution strategies to enhance microbial tolerance. Feedstock optimization and waste valorization explore lignocellulosic biomass, agricultural waste, and industrial by-products alongside microbial consortia for efficient bioconversion of complex substrates. Process automation and AI-driven optimization integrate machine learning and AI to refine fermentation parameters and develop real-time monitoring systems for enhanced process control. For simplified recovery, cost-effective downstream processing focuses on solvent-free extraction techniques to minimize environmental impact and investigate self-flocculating and autolytic bacterial strains. Sustainability and commercialization efforts assess life cycle impacts to enhance environmental benefits while establishing scalable bioprocesses to reduce costs and compete with petroleum-based plastics.

PHA production from halophiles presents several challenges, including high salinity requirements, which complicate large-scale fermentation and downstream processing. Maintaining optimal growth conditions requires specialized bioreactors resistant to corrosion and salt accumulation. Also, halophilic cultures often exhibit slower growth rates and lower biomass yields than mesophilic counterparts. The presence of intracellular salts in harvested biomass can hinder PHA purification, necessitating additional washing steps. Moreover, the economic viability of halophilic PHA production remains a concern due to the costs associated with maintaining extreme conditions.

Future directions include metabolic engineering to enhance PHA accumulation and cell growth under moderate salinity, reducing process complexity. Optimizing nutrient formulations and developing cost-effective halophilic feedstocks can improve productivity. Additionally, advances in continuous bioprocessing and integrated recovery methods, such as membrane filtration, can enhance efficiency. Exploring novel halophilic strains with superior PHA yield and stress tolerance will further improve sustainability and commercial feasibility.

## 8. Conclusions

PHAs hold immense promise as biodegradable alternatives to conventional plastics; however, their large-scale production remains limited by high costs and technical challenges. Traditional PHA production relies on pure microbial cultures and refined carbon sources, which makes it economically unfeasible. The emergence of NGIB represents a paradigm shift by leveraging extremophilic microbes, such as halophiles and thermophiles, as a robust chassis for cost-effective and sustainable bioprocesses. These microorganisms naturally thrive in extreme environments that suppress microbial contamination, reducing the need for costly sterilization and stringent process control. Advancements in metabolic engineering, synthetic biology, and feedstock diversification have enhanced the efficiency of PHA production. Utilizing alternative carbon sources, such as biowaste, syngas, methane, and CO_2_, reduces the dependence on expensive refined sugars. Replacing freshwater with seawater or treated wastewater significantly lowers operational costs and environmental impacts. PHA production can be economically viable by integrating continuous culture systems with energy-efficient bioreactors. Future research should optimize metabolic pathways and fermentation strategies to ensure scalable industrial implementation, ultimately positioning PHA as a competitive and sustainable alternative to petroleum-based plastics.

## Figures and Tables

**Table 1:** Diversity of halophilic archaeal microbes to produce polyhydroxyalkanoates.

PHA Producer	Substrate (%)	Salt (NaCl) (%)	Reactor	Cell Dry Mass (g/L)	Polyhydroxyalkanoate (PHA)			References
					Composition (%mol Copolymer)	Content (wt%)	Yield (g/L)	
Pure Sugars								
*Haloarcula marismortui* ATCC 43049 (33960/pWLEC)	Glucose (2)	20	Shake flask	15.4	P(3HB)	18	2.77	[47]
*Haloferax mediterranei*	Glucose ^a^ (1)	20	Fed-batch	85.8	P(3HB-3HV) [10.7 mol% 3HV]	48.6	41.69	[48]
*Halogranum amylolyticum* TNN58	Glucose ^a^ (1)	20	Fed-batch (7.5 L)	5.4	P(3HB-3HV) [20.1 mol% 3HV]	26.6	1.4	[50]
*Halogeometricum borinquense* E3	Glucose ^a^ (2)	20	Shake flask	2.1	P(3HB-3HV) [21.47 mol% 3HV]	73.51	1.54	[51]
*Natrinema ajinwuensis* RM-G10	Glucose ^a^ (1)	20	Shake flask (Repeat batch)	24.2	P(3HB-3HV) [13.93 mol% 3HV]	61	14.78	[52]
Biowastes								
Starch-based feedstocks								
*H. mediterranei*	Extruded starch (1)	23.4	Shake flask	39.4	P(3HB-3HV) [10.4 mol% 3HV]	50.8	20/01	[53]
	Extruded rice bran: corn starch::1:8) (1)	23.4	Fed-batch	140	P(3HB-3HV) [10.4 mol% 3HV]	55.6	77.84	[54]
	Extruded corn starch (1)	23.4	Fed-batch	62.6	P(3HB-3HV) [10.4 mol% 3HV]	38.7	24.2	
*H. mediterranei* CGMCC 1.2087	Starch (1) + AS-168 medium	20	Shake flask	7.33	P(3HB-3HV) [9.33 mol% 3HV]	18.21	1.33	[49]
	Starch (1) + MST medium	20	Shake flask	7.01	P(3HB-3HV) [13.37 mol% 3HV]	24.88	1.74	
*Natrinema* sp. 1KYS1	Starch (2)	25	Shake flask	2.21	P(3HB-3HV)	2.48	0.055	[46]
	Corn starch (2)	25	Shake flask	0.17	P(3HB-3HV) [25 mol% 3HV]	53.14	0.075	
Dairy and ethanol industry waste								
*H. mediterranei* DSM 1411	Hydrolyzed whey	15.6	Shake flask	24	P(3HB-3HV) [8 mol% 3HV]	50	12	[55]
	Hydrolyzed whey sodium valerate and γ-butyrolactone	15	Batch	16.8	P(3HB-3HV-4HB) [21.8 mol% 3HV; 5.1 mol% 4HB]	87.5	14.7	[56]
	Pre-treated vinasse (25%)	20	Shake flask	28.14	P(3HB-3HV) [12.36 mol% 3HV]	70	19.7	[57]
	Pre-treated vinasse (50%)	20	Shake flask	26.34	P(3HB-3HV) [14.09 mol% 3HV]	66	17.4	
	Rice-based ethanol stillage	20	Shake flask	23.12	P(3HB-3HV) [15.4 mol% 3HV]	71	16.42	[58]
*H. marismortui* MTCC 1596	Raw vinasse (10)	20	Shake flask	12	P(3HB)	23	2.8	[59]
	Raw vinasse (pre-treated—activated carbon) (100)	20	Shake flask	15	P(3HB)	30	4.5	
*Natrinema* sp. 1KYS1	Whey (2)	25	Shake flask	0.454	P(3HB-3HV)	19.92	0.091	[46]
Agro-industrial waste and other feedstocks								
*Natrinema* sp. 1KYS1	Melon waste (2)	25	Shake flask	0.37	P(3HB-3HV)	10.5	0.039	[46]
	Apple waste (2)	25	Shake flask	2.55	P(3HB-3HV)	3.02	0.077	
	Tomato waste (2)	25	Shake flask	3.85	P(3HB-3HV)	12.03	0.46	
*H. mediterranei*	Olive mill wastewater (15)	22	Shake flask	10	P(3HB-3HV) [6.5 mol% 3HV]	43	4.3	[60]
*Halogeometricum borinquense* E3	Sugarcane bagasse hydrolysate (20)	20	Shake flask	3.17	P(3HB-3HV) [13.29 mol% 3HV]	50.4	1.59	[61]
	Starch (2)	20	Shake flask	6.2	P(3HB-3HV) [13.11 mol% 3HV]	74.19	4.6	[62]
	Cassava waste hydrolysate (10)	20	Shake flask	3.4	P(3HB-3HV) [19.65 mol% 3HV]	44.7	1.52	

a: Precursor added. P(3HB): poly(3-hydroxybutyrate); P(3HB-3HV): poly(3-hydroxybutyrate-3-hydroxyvalerate).

**Table 3:** A comparison of the archaeal and bacterial polyhdroxyalkanoate-producing potential from biowastes.

Criteria	Archaea	Bacteria
Max. PHA Yield (g/L)	77.8 (Rice bran-corn starch mix)	10.5 (Optimized crude glycerol)
Max. PHA Content (%)	87.5	95.26
Best Feedstocks	Hydrolyzed whey, rice bran-corn starch, ethanol waste	Fructose syrup, sugar beet molasses, crude glycerol
Copolymer Composition	21.8 mol% 3HV, 5.1 mol% 4HB	52 mol% 3HV, high M.Wt. PHA
Waste Valorization	Strong (vinasse, bagasse, agro-waste)	Moderate (biodiesel byproducts, wheat straw)
Industrial Feasibility	More established (high yield and adaptability to hypersaline environments)	More cost-effective (low-cost substrates, non-sterile fermentation)

## Data Availability

Not applicable.

## Abbreviations

The following abbreviations are used in this manuscript:

PHA	Polyhydroxyalkanoates
NGIB	Next-Generation Industrial Biotechnology
scl-PHA	Short-chain-length polyhydroxyalkanoate
mcl-PHA	Medium-chain-length polyhydroxyalkanoate
P(3HB)	Poly(3-hydroxybutyrate)
P(3HB-3HV)	Poly(3-hydroxybutyrate-3-hydroxyvalerate)
CDW	Cell dry weight
M.Wt.	Molecular weight
SCB	Sugarcane bagasse
NADH/NAD+	Nicotinamide adenine dinucleotide hydride/Nicotinamide adenine dinucleotide
CRISPR/Cas9	Clustered regularly interspaced short palindromic repeats Cas protein systems
CRISPRi	Clustered regularly interspaced short palindromic repeats interference
